# Using multiple genetic variants as instrumental variables for modifiable risk factors

**DOI:** 10.1177/0962280210394459

**Published:** 2012-06

**Authors:** Tom M Palmer, Debbie A Lawlor, Roger M Harbord, Nuala A Sheehan, Jon H Tobias, Nicholas J Timpson, George Davey Smith, Jonathan AC Sterne

**Affiliations:** 1 MRC Centre for Causal Analyses in Translational Epidemiology, School of Social and Community Medicine, University of Bristol, Bristol, UK; 2 School of Social and Community Medicine, University of Bristol, Bristol, UK; 3 Departments of Health Sciences and Genetics, University of Leicester, Leicester, UK; 4 School of Clinical Sciences, University of Bristol, Bristol, UK

**Keywords:** causal inference, econometrics, epidemiology, genetics, instrumental variables, Mendelian randomisation

## Abstract

Mendelian randomisation analyses use genetic variants as instrumental variables (IVs) to estimate causal effects of modifiable risk factors on disease outcomes. Genetic variants typically explain a small proportion of the variability in risk factors; hence Mendelian randomisation analyses can require large sample sizes. However, an increasing number of genetic variants have been found to be robustly associated with disease-related outcomes in genome-wide association studies. Use of multiple instruments can improve the precision of IV estimates, and also permit examination of underlying IV assumptions. We discuss the use of multiple genetic variants in Mendelian randomisation analyses with continuous outcome variables where all relationships are assumed to be linear. We describe possible violations of IV assumptions, and how multiple instrument analyses can be used to identify them. We present an example using four adiposity-associated genetic variants as IVs for the causal effect of fat mass on bone density, using data on 5509 children enrolled in the ALSPAC birth cohort study. We also use simulation studies to examine the effect of different sets of IVs on precision and bias. When each instrument independently explains variability in the risk factor, use of multiple instruments increases the precision of IV estimates. However, inclusion of weak instruments could increase finite sample bias. Missing data on multiple genetic variants can diminish the available sample size, compared with single instrument analyses. In simulations with additive genotype-risk factor effects, IV estimates using a weighted allele score had similar properties to estimates using multiple instruments. Under the correct conditions, multiple instrument analyses are a promising approach for Mendelian randomisation studies. Further research is required into multiple imputation methods to address missing data issues in IV estimation.

## 1 Introduction

Mendelian randomisation analyses use genetic variants as instrumental variables (IVs) to make causal inferences about the effect of modifiable risk factors on health- and disease-related outcomes in the presence of unobserved confounding of the relationship of interest.^1^^–^^5^ Use of Mendelian randomisation is growing rapidly.^4^^–^^7^ However, using genetic variants as IVs poses statistical challenges.^5,8^^–^^11^ In particular, there is a need for large sample sizes because of the relatively small proportion of variation in risk factors typically explained by genetic variants.^5,12,13^

Recent decreases in genotyping costs and increases in genome-wide association studies (GWAS), have facilitated discovery of a substantial number of genetic variants associated with risk factors and disease-related outcomes, such as adiposity^14^^–^^16^ and type 2 diabetes.^17^^–^^27^ Consideration of multiple instruments for Mendelian randomisation applications is therefore timely due to increasing availability of suitable variants. In this article we discuss the use of multiple genetic variants as IVs, both for increasing statistical precision and for testing underlying IV assumptions.

The structure of the article is as follows: we describe instrumental variable assumptions (Section 1.1) and introduce an illustrative Mendelian randomisation analysis and present separate IV estimates for four instruments (Section 2). We then discuss the use of multiple instruments to help address some of the genetic and statistical issues that can affect Mendelian randomisation analyses (Sections 3 and 4), including the results of simulation studies (Section 5). We return to the example and simulation to compare IV estimates using multiple instruments and allele scores (Section 6), assess the impact of missing data (Section 6.2) and discuss the implications of our findings (Section 7).

### 1.1 Instrumental variable assumptions

An IV (instrument) *G* is defined as a variable that satisfies the following assumptions:

1. *G* is associated with the risk factor (phenotype or intermediate variable) of interest *X*;
2. *G* is independent of the (unobserved) confounding factors *U* of the association between *X* and the outcome *Y*;
3. *G* is independent of outcome *Y* given *X* and *U*.

In the context of Mendelian randomisation, these assumptions can be expressed as: genotype is associated with the modifiable risk factor of interest (assumption 1); genotype is independent of unmeasured confounding factors that could bias conventional epidemiological associations between the risk factor and the outcome (assumption 2); genotype is related to the outcome only via its association with the risk factor (assumption 3). The second assumption can be justified through Mendel’s laws when applied to independent heritable units.^5,28^

If we further assume that intervention on the risk factor only affects the value of the risk factor, and hence affects the outcome only through this induced change in the risk factor, then the IV assumptions imply the ‘exclusion restriction’^11,29^ and its weaker form known as ‘conditional mean independence’ (used in structural mean models).^30^ This additional assumption allows causal inferences to be drawn from IV analyses.

## 2 Illustrative Mendelian randomisation analysis: single instrument estimates

Our example investigates the causal effect of fat mass on bone mineral density (BMD) using four genotypes known to be associated with adiposity from previous GWAS. A previous study found a positive effect of fat mass on BMD using SNPs associated with the *FTO* and *MC4R* genes as IVs.^31^ The authors concluded that higher fat mass caused increased accrual of bone mass in childhood. We consider whether the IV estimates from the separate instruments are of similar magnitude; whether use of multiple instruments increases the precision of IV estimates; the use of allele scores as IVs; and the impact of missing data on IV estimates.

### 2.1 Data

Our example uses data from the Avon Longitudinal Study of Parents and Children (ALSPAC).^32^ ALSPAC is a longitudinal, population-based birth cohort study that recruited 14 541 pregnant women resident in Avon, UK, with expected dates of delivery 1 April 1991 to 31 December 1992 (http://www.alspac.bris.ac.uk).^32^ Out of this 13 988 live born infants survived to at least one year of age. Children eligible for inclusion in our analysis: (1) had DNA available for genotyping; (2) attended the research clinic at age 9 and (3) had complete data on height and dual energy X-ray densitometry (DXA) scan-determined total fat mass and total BMD.

### 2.2 Selection of genotypes

Eleven adiposity-related SNPs identified in previous GWAS have been genotyped in ALSPAC. For these analyses we decided *a priori* to use the four SNPs, namely *FTO* (rs9939609), *MC4R* (rs17782313), *TMEM18* (rs6548238) and *GNPDA2* (rs10938397), that had the strongest associations with adiposity in previous studies.^14^^–^^16^ Functional studies are required to ascertain the specific biological pathways through which these polymorphisms affect adiposity. Whilst most pathways to greater adiposity are likely to involve influences on diet/appetite or physical activity, here for the assessment of the IV assumptions (Section 3) we assume that the underlying mechanisms by which they influence diet or physical activity differ for each of the variants under consideration. Although current knowledge about their function is limited, their location on different chromosomes suggests that their influences may indeed be independent.^14^^–^^16,33,34^

**Figure 1.**
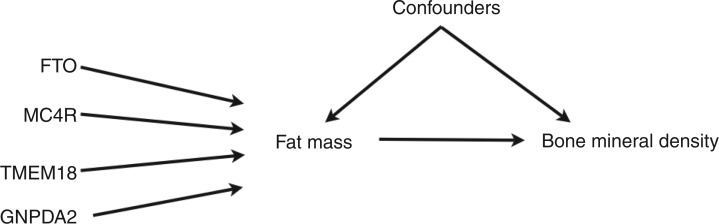
DAG for a Mendelian randomisation analysis using four genetic variants as instrumental variables for the effect of fat mass on bone mineral density.

The IV assumptions can be uniquely encoded in a directed acyclic graph (DAG).^11^ The proposed DAG for our examplar multiple instrument model is shown in Figure 1.

### 2.3 Statistical methods

Fat mass and BMD were positively skewed and were log transformed. To account for sex and age differences in fat mass and BMD, age and sex standardised z-scores of log transformed fat mass and BMD were used in the analysis. Genotypes were incorporated into IV models assuming an additive genetic model for the genotypes coded 0, 1 and 2, as shown in Table 1. Height and height-squared were included as covariates in analyses. We exponentiated parameter estimates to derive ratios of geometric mean BMD per standard deviation (SD) increase in log fat mass. Analyses were performed in Stata 11.0.

**Table 1.** Study participant characteristics, total eligible children *N* = 5509

	*N* (%)	Mean (SD), geometric mean (95% CI) or *N* (%)	HWE *p*-value for genotypes
Gender: *N*(%) Female	5509 (100%)	2713 (49.3%)	
Age: Mean (SD) years	5509 (100%)	9.88 (0.32)	
BMD: geometric mean (95% CI) g/cm^2^	5509 (100%)	0.902 (0.900, 0.903)	
Fat mass: geometric mean (95% CI) g	5509 (100%)	7209 (7100, 7320)	
Height: mean (SD) cm	5509 (100%)	139.6 (6.3)	
*FTO* (rs9939609):	5091 (92%)	TT = 0: 868 (37%)	0.51
		TA = 1: 2413 (47%)	
		AA = 2: 810 (16%)	
*MC4R* (rs17782313):	5412 (98%)	TT = 0: 3115 (58%)	0.04
		TC = 1: 2017 (37%)	
		CC = 2: 280 (5%)	
*TMEM18* (rs6548238):	5323 (97%)	CC = 0: 3705 (70%)	0.57
		CT = 1: 1465 (28%)	
		TT = 2: 153 (3%)	
*GNPDA2* (rs10938397):	5303 (96%)	AA = 0: 1731 (33%)	0.84
		AG = 1: 2604 (49%)	
		GG = 2: 968 (18%)	

HWE: Hardy–Weinberg Equilibrium.

IV estimation used the two-stage least squares (TSLS) estimator implemented in the user written Stata command ivreg2.^35^^–^^37^ The Hausman test of endogeneity^38^ was used to compare the difference between the ordinary-least-squares (OLS) and TSLS estimates using the user-written Stata command ivendog.^35^ (In econometrics a risk factor affected by unmeasured confounding factors, such that the assumptions of linear regression are violated, is termed an endogenous variable.) In models including multiple instruments the Sargan test of over-identification (discussed in Section 4.1), available in the ivreg2 command, was used to test the joint validity of the instruments.^39^

### 2.4 Results for separate instruments

Table 1 shows characteristics of the 5 509 eligible children. Of these, 5 091 (92%) had valid genotype data for *FTO*, 5,412 (98%) for *MC4R*, 5,323 (97%) for *TMEM18*, 5 303 (96%) for *GNPDA2* and 4 796 (87%) for all four SNPs. Mean age at the time of the DXA scans was 9.9 years. There was no strong evidence against the *FTO*, *TMEM18* and *GNPDA2* genotypes being in Hardy–Weinberg equilibrium. The *MC4R* genotypes had an Hardy–Weinberg equilibrium *p*-value of 0.04 in our sample, though in the whole ALSPAC cohort the corresponding *p*-value was 0.1.

Table 2 shows that there is no strong evidence of associations of the *FTO*, *MC4R* or *GNPDA2* with height, lean mass, mother’s educational achievement and head of household social class. There is some evidence for these data that *TMEM18* is associated with lean mass and mother’s educational achievement. Under the IV assumptions, *TMEM18* genotypes only affect BMD through fat mass; so for now we view these latter two associations as chance findings similar to baseline covariates found to be associated with treatment group in a randomised controlled trial (RCT).

**Table 2.** Associations of genotypes with potential confounding factors

		Number of risk alleles			
Genetic variant Covariate (unit) (*N*)		0	1	2	
Continuous confounding factors		Mean (95% CI)	Mean (95% CI)	Mean (95% CI)	Regression coefficient* (95% CI), *p*-value
*FTO*	Height (cm) (5091)	139.5 (139.2, 139.7)	139.6 (139.3, 139.8)	139.8 (139.4, 140.3)	0.18 (−0.07, 0.42), *p* = 0.165
	Lean mass (g) (2515)	24 426 (24 218, 24 634)	24 620 (24 439, 24 800)	24 593 (24 287, 24 899)	104 (−74, 283), *p* = 0.253
*MC4R*	Height (cm) (5412)	139.7 (139.4, 139.9)	139.5 (139.2, 139.8)	140.1 (139.4, 140.9)	0.01 (−0.28, 0.29), *p* = 0.965
	Lean mass (g) (2685)	24 548 (24 387, 24 708)	24 636 (24 438, 24 834)	24 910 (24 362, 25 458)	128 (−78, 334), *p* = 0.222
*TMEM18*	Height (cm) (5323)	139.7 (139.5, 139.9)	139.5 (139.1, 139.8)	139.3 (138.3, 140.3)	−0.24 (−0.56, 0.08), *p* = 0.137
	Lean mass (g) (2640)	24 770 (24 622, 24 917)	24 286 (24 053, 24 519)	24 017 (23 293, 24 740)	−447 (−679, −215), *p* < 0.001
*GNPDA2*	Height (cm) (5303)	139.5 (139.3, 139.8)	139.6 (139.4, 139.9)	139.7 (139.3, 140.1)	0.10 (−0.14, 0.34), *p* = 0.420
	Lean mass (g) (2625)	24 596 (24 382, 24 810)	24 655 (24 479, 24 832)	24 525 (24 234, 24 816)	−21 (−198, 155), *p* = 0.812
Categorical confounding factors		*n*/*N* (%)	*n*/*N* (%)	*n*/*N* (%)	Odds ratio* (95% CI), *p*-value
*FTO*	MEA (2421)	139/857 (16%)	189/1161 (16%)	69/403 (17%)	1.03 (0.88, 1.20), *p* = 0.726
	HHSC (2329)				Chi-squared *p* = 0.038
*MC4R*	MEA (2591)	255/1492 (17%)	155/971 (16%)	25/128 (20%)	0.99 (0.83, 1.18), *p* = 0.929
	HHSC (2485)				Chi-squared *p* = 0.432
*TMEM18*	MEA (2543)	314/1765 (18%)	107/705 (15%)	4/73 (5%)	0.74 (0.60, 0.92), *p* = 0.006
	HHSC (2438)				Chi-squared *p* = 0.556
*GNPDA2*	MEA (2532)	151/838 (18%)	203/1236 (16%)	69/458 (13%)	0.90 (0.77, 1.04), *p* = 0.159
	HHSC (2432)				Chi-squared *p* = 0.754

MEA: Mother’s highest educational achievement is a binary variable derived from the groups 0 = CSE, O-level, Vocational and 1 = A-level and degree.

HHSC: Head of household social class coded as categorical variable I, II, III non-manual, III manual, IV and V.

* Assuming an additive genetic model.

Table 3 shows OLS and IV estimates of the effect of fat mass on BMD in children with complete data. The OLS estimate of the ratio of geometric means per SD increase in log fat mass (adjusted for height and height-squared but not other potential confounders) was 1.22 (95% CI: 1.19, 1.26). The IV estimates of the ratio of geometric means, using each SNP separately, varied between 0.98 (95% CI 0.47–2.03) for *GNPDA2* and 2.33 (1.34–4.05) for *MC4R*. These four IV estimates generally suggest that BMD has a positive effect on fat mass, although the lower limit of the confidence interval for the *TMEM18* estimate and both the lower limit of the confidence interval and point estimate using *GNPDA2* as an instrument, were less than 1. For *MC4R* and *TMEM18*, there was evidence that the IV estimate differed from the OLS estimate, based on the Hausman test of endogeneity (*p*-values 0.006 and 0.089, respectively), with both suggesting a stronger positive association than that found in the OLS analysis.

**Table 3.** OLS and IV estimates of the effect of fat mass on bone mineral density (BMD) based on complete case analysis, *N* = 4796^a^

Method	First stage regression coefficient (95% CI)	First stage *R^2^*	First stage * F*-statistic	Ratio of geometric mean BMD^b^ (95% CI)	SE of estimate (log scale)	Hausman test *p*-value	Sargan test P-value
OLS	NA	NA	NA	1.22 (1.19, 1.26), *p* < 0.001	0.014	NA	NA
IV: SNP(s) used as IV							
*FTO*	0.11 (0.08, 0.15)	0.0082	39.83	1.44 (1.05, 1.97), *p* = 0.024	0.16	0.300	NA
*MC4R*	0.09 (0.05, 0.13)	0.0037	17.85	2.33 (1.34, 4.05), *p* = 0.003	0.28	0.006	NA
*TMEM18*	−0.06 (−0.11, −0.02)	0.0016	7.47	2.27 (0.98, 5.28), *p* = 0.056	0.43	0.089	NA
*GNPDA2*	0.05 (0.01, 0.09)	0.0016	7.57	0.98 (0.47, 2.03), *p* = 0.953	0.37	0.540	NA
*FTO*, *MC4R*	NA	0.0119	29.92	1.67 (1.27, 2.19), *p* < 0.001	0.14	0.020	0.11
*FTO*, *MC4R*, *TMEM18*	NA	0.0136	21.95	1.73 (1.34, 2.24), *p* < 0.001	0.13	0.010	0.22
*FTO*, *MC4R*, *TMEM18*, *GNPDA2*	NA	0.0153	18.59	1.63 (1.28, 2.06), *p* < 0.001	0.12	0.013	0.16
Unweighted allele score (4 SNPs)	0.06 (0.04, 0.08)	0.0069	33.15	1.40 (0.99, 1.98), *p* = 0.055	0.18	0.430	NA
Weighted allele score (4 SNPs)	0.19 (0.15, 0.24)	0.0153	74.35	1.63 (1.29, 2.07), *p* < 0.001	0.12	0.012	NA

a Analyses adjusted for height and height squared.

b For a 1 unit increase in *z*-score of age and gender standardised fat mass.

The first stage *R*^2^ and *F*-statistics for the instruments based on the explained variation in standardised log fat mass show the expected ranking, with *FTO* genotype explaining the largest proportion of variation followed by *MC4R*, *TMEM18* and *GNPDA2* (these latter two genotypes explained approximately equal variation). The variation in standardised log fat mass explained by each SNP was small, ranging from 0.16% to 0.80%, and the *TMEM18* and *GNPDA2* SNPs were weak instruments, based on their first-stage *F*-statistic being less than 10 (Section 4.2). Consistent with the proportion of variation in fat mass explained by each SNP, the standard error (SE) of the IV estimate was smallest for the IV estimate using the *FTO* SNP (0.16) and largest for *TMEM18* and *GNPDA2* SNPs (0.43 and 0.37). IV estimates using multiple instruments are described in Section 6.

## 3 Using multiple instruments to address potential biases in Mendelian randomisation analyses

Population stratification, linkage disequilibrium and pleiotropy have been identified as factors that could bias Mendelian randomisation analyses.^2,5,11,40^ We briefly describe them, and the use of multiple instruments to address issues they raise.

### 3.1 Population stratification

Population stratification occurs when a sample is composed of a mixture of populations and so contains latent ancestral structure. If there are corresponding differences in the prevalence of the outcome of interest by this structure, then genotype-risk factor associations may result from the presence of ancestrally informative alleles rather than biological function.^41^ Some genetic variants that are potential candidates for use as IVs in Mendelian randomisation studies could have been influenced by such population stratification.^5,42^^–^^45^ Population stratification therefore has the potential to bias estimates of causal effects in Mendelian randomisation studies.^5^

### 3.2 Linkage disequilibrium

Linkage disequilibrium (LD) is correlation between allelic states at different loci on a stretch of the same chromosome when assessed within a population. LD is a function of the frequency of recombination and is subject to regional genomic characteristics as well as more stochastic processes which may be influenced by the physical distance between two loci as well as the relative age of the population in question. Extensive LD can increase the statistical power of a study to detect genotype-risk factor associations and is exploited in GWAS studies where an LD-based set of tag SNPs is chosen to maximise the amount of genetic variation captured per SNP.^46,47^ SNPs that are associated with phenotypes in GWAS are unlikely to be functional variants, but rather to be in LD with the unknown functional variant(s).^46,47^ IV assumptions are not violated when tag SNPs are used as IVs, providing that they are in LD only with the functional variant(s).^5,11^ However, if tag SNPs are also in LD with a variant that affects the outcome of interest via a pathway that does not include the risk factor of interest the IV assumptions will be violated.^5^

### 3.3 Pleiotropy

Pleiotropy refers to a single gene having multiple biological functions. In the context of Mendelian randomisation analyses, SNPs in or near genes with pleiotropic effects that directly or indirectly influence the outcome other than through the risk factor of interest violate the IV assumptions.^11^ In our example, if any of the adiposity variants had effects on pathways that influence BMD other than through adiposity, for example, if they influenced calcium or vitamin D metabolism, then IV assumptions would not hold.

### 3.4 Use of multiple instruments

Population stratification and pleiotropy can to some extent be dealt with by using ethnically homogenous study populations, identifying and incorporating population strata in the analysis and ensuring that the function of the genetic instrument is well understood.^5^ Comparison of IV estimates based on multiple genetic variants with independent effects on the risk factor of interest provides an additional way to identify bias resulting from these issues. If IV estimates from different variants are similar, it is less plausible that LD or pleiotropy are present.

Comparison of IV estimates from independent genetic variants is analogous to comparing the results of RCTs of different classes of blood pressure lowering drugs, which lower blood pressure by different mechanisms. If the effect of the drug on stroke risk in each RCT is proportional to the direction and magnitude of its effect on blood pressure, this strengthens the evidence for a causal link between blood pressure and stroke risk, and against the drugs having effects on stroke risk through other mechanisms. Such consistency would also argue against the possibility that the trials were affected by methodological flaws that biased their results.

It is possible that separate IV estimates could be identical but biased to a similar extent by population stratification, because stochastic- or selection-driven non-independence that is not predicted by LD profiles could influence more than one genetic variant that affects a given risk factor. Databases such as dbSNP (http://www.ncbi.nlm.nih.gov/projects/SNP/) that provide the fixation index *F_ST_* (a measure of population differentiation), or equivalent information, can be used to examine population stratification..

## 4 Statistical issues relating to use of multiple instruments in Mendelian randomisation analyses

### 4.1 Over-identification

Over-identification refers to the situation when there is more than one instrument for a single risk factor of interest or, more generally, when there are more instruments than endogenous variables. In such circumstances testing the ‘over-identification restriction’ checks the joint validity of multiple instruments by testing whether they give the same estimates when used singly or in linear combination. There are two commonly used tests of over-identification; the Hansen test and the Sargan test.^39,48^ Rejection of an over-identification test is taken to indicate that at least one of the instruments is not valid (i.e., it does not give the same estimate as the other instruments).^49^

Verifying that the genotypes are independent of the measured confounding factors (Table 2) is an indication of the validity of the instruments.^50^ However, genotypes could still be associated with unmeasured confounders.

### 4.2 Finite sample bias and instrument strength

IV estimators such as TSLS are asymptotically unbiased but biased in finite samples, with such bias inversely proportional to the amount of phenotypic variability explained by the instrument.^51^ Two closely related measures of this are the first-stage regression *F*-statistic and coefficient of determination *R*^2^. It is important to report these. If measured confounders are included then the partial *R*^2^ and *F*-statistics for the instruments should be reported.^52^

In Mendelian randomisation the first stage *R*^2^ is the proportion of risk factor variability explained by genotype. The relationship between the *F* and *R*^2^ statistics is given by:
(1)
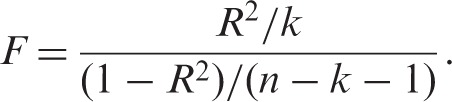

where *k* is the number of parameters in the model (in this case instruments). The relative bias of the TSLS estimator to the OLS estimator is related to the inverse of the *F*-statistic.^53^ Hahn and Hausman gave a simplified version of the relative bias as approximately the inverse of the *F*-statistic:^54^^–^^56^
(2)
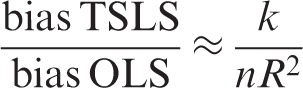



As *R*^2^ increases the relative bias of TSLS decreases, but including additional instruments that do not increase the first stage *R*^2^ increases the relative bias of TSLS. A first stage *F*-statistic less than 10 is often taken to indicate a weak instrument, although this is not a strict limit but a rule of thumb drawn from simulation studies.^53,57,58^ Equation (2) shows that *F* = 10 corresponds to approximately 10% relative bias.^54,58^ Alternative IV estimators to TSLS may have better finite sample properties when instruments explain a small proportion of phenotypic variability.^59,60^

### 4.3 Statistical power

Genotypic effects on phenotypes are typically small, so Mendelian randomisation analyses can require very large sample sizes to obtain adequate power.^5,13^ When multiple instruments are used in the TSLS estimator, the resulting IV estimate can be viewed as the efficient linear combination of the separate IV estimates.^61^ Provided that each instrument is valid, use of multiple instruments will increase the precision of the IV estimate compared with the separate IV estimates.^61^ Donald and Newey investigated the trade off for multiple instruments where increasing precision can also increase bias, and suggested using the instruments that minimise an approximate mean squared error (MSE) criterion.^62^ Pierce et al. recently estimated the power of Mendelian randomisation studies in a range of settings, using both single and multiple genetic instruments.^13^

In studies where genetic data are not obtained from GWAS (in which imputation based on LD is typically performed) there are typically some missing observations for each genetic variant, due to failure of genotyping or ambiguous genotype allocation. Missing data typically occur in different individuals for each variant. They can therefore result in a considerable cumulative reduction in the number of individuals with complete data on all genotypes, and hence reduce the power of multiple instrument Mendelian randomisation analyses. One approach to dealing with missing data is multiple imputation.^63^ Whilst there has been considerable research into methods of imputation we are not aware of specific research into appropriate multiple imputation models for IV estimation.

### 4.4 Use of an allele score as an instrumental variable

An allele score is a weighted or unweighted sum of the number of ‘risk’ alleles across several genotypes: weights are usually based on each genotype’s effect on the phenotype. Use of such scores is becoming more common in gene–disease association studies.^64^^–^^66^ To justify the use of an allele score the genotypes should have an approximately additive effect on the risk factor. For an unweighted score they should also have similar per allele effects.

The use of an allele score as a single IV, compared with multiple instruments, will cause the first stage *F*-statistic to increase, since the number of parameters in the model is reduced. Therefore, the relative bias of the TSLS estimator to the OLS estimator will decrease. However, if the weights are estimated from the same data in which the score is used as an instrument then the single degree of freedom for the allele score *F*-statistic may not be appropriate. When using an allele score the IV estimator is exactly identified, because there is a single instrument and single phenotype, and it is therefore not possible to use an over-identification test for the joint validity of the SNPs.

In general, using an unweighted allele score will have lower power than the multiple instrument approach, since the latter will estimate the efficient linear combination of the genotypes.^61^ Given appropriate weighting, results from IV analyses using weighted allele scores will be similar to the multiple instruments approach.

## 5 Multiple instrument simulations

We investigated the use of multiple instruments through two simulations both based on our example. Specifically, we investigated bias and precision of IV estimates including: (i) additional non-weak instruments and (ii) weak instruments.

### 5.1 Simulation 1: non-weak instruments

Data were simulated as follows, where *G*_1_, *G*_2_ and *G*_3_ are genotype variables coded additively, *X* is the risk factor, *Y* the disease outcome, *U* the unmeasured confounder and subscript *i* denotes a subject:

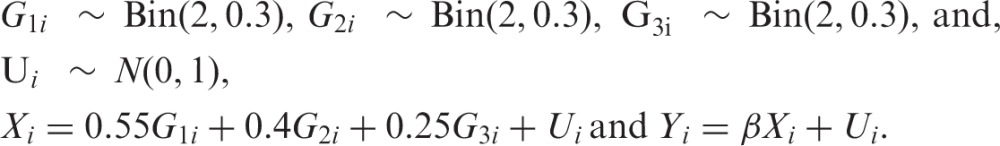



The values of the coefficients on the genotypes were chosen so that *G*_1_ explained the most variability in *X*, followed by *G*_2_ and *G*_3_. The value of the causal effect of *X* on *Y,* *β*, was set to 1. We monitored the estimates of *β* from the following models:

1. OLS estimate of the regression of *Y* on *X,*
2. TSLS using *G*_1_ as the instrument,
3. TSLS using *G*_1_ and *G*_2_ as instruments,
4. TSLS using *G*_1_–*G*_3_ as instruments,
5. TSLS using an unweighted allele score of *G*_1_–*G*_3_ as an instrument,
6. TSLS using a weighted allele score of *G*_1_–*G*_3_ as an instrument.

We used 10 000 replications, each with a sample size of 5 000 observations. Weighted allele scores were generated by summing each genotype multiplied by its estimated coefficient from the linear regression of the risk factor on that particular genotype, divided by the sum of weights. We derived the average bias, MSE, average SE of the IV estimates, coverage, average *R*^2^ and *F*-statistics and average absolute TSLS/OLS bias ratio (see Equation (2) in Section 4.2). In a further study we plotted the power curves for models 2–6 for the Wald test of the null hypothesis that *β*_ _= 1. For this we used 10 000 replications for values of *β* in the range 0.8–1.2.

### 5.2 Simulation 1: results

Table 4 shows that the average *R*^2^ values for *G*_1_, *G*_1_ and *G*_2_ and *G*_1_–*G*_3_ were 0.12, 0.19 and 0.22, respectively. The average SE decreased by 20% with the inclusion of *G*_2_ and by a further 6% with the inclusion of *G_3_*.

**Table 4.** Simulation 1 (non-weak instruments): results (Monte Carlo standard error reported in brackets beside each estimate)

Model	Average bias	MSE	Average SE	Coverage	Average *R^2^*	Average F	Average absolute TSLS/OLS bias ratio
1. OLS	0.8194 (0.00005)	0.6714 (0.00009)	0.0054 (7 E–7)	0	NA	NA	NA
2. TSLS *G*_1_	−0.0019 (0.0004)	0.0016 (0.00002)	0.03991 (0.00003)	0.9523 (0.0021)	0.1163 (0.0001)	581.41 (0.504)	0.0022 (0.0005)
3. TSLS *G*_1_ & *G*_2_	−0.00004 (0.0003)	0.0010 (0.00002)	0.03215 (0.00002)	0.9467 (0.0022)	0.1898 (0.0001)	474.09 (0.333)	0.0001 (0.0004)
4. TSLS *G*_1_–*G*_3_	0.00084 (0.0003)	0.0009 (0.00001)	0.0301 (0.00002)	0.9487 (0.0022)	0.2212 (0.0001)	368.41 (0.243)	0.0012 (0.0004)
5. TSLS allele score *G*_1_–*G*_3_	−0.00098 (0.0003)	0.0010 (0.00002)	0.0316 (0.00002)	0.9486 (0.0022)	0.1981 (0.0001)	990.22 (0.685)	0.0010 (0.0004)
6. TSLS weighted allele score *G*_1_–*G*_3_	0.00084 (0.0003)	0.0009 (0.00001)	0.0301 (0.00002)	0.9492 (0.0022)	0.2212 (0.0001)	1105.43 (0.730)	0.0012 (0.0004)

MSE: mean squared error, SE: standard error, TSLS: two-stage least squares, OLS: ordinary least squares.

Models 4 and 6, (multiple instruments using the three genotypes and weighted allele score), had almost identical properties and had the smallest MSE. Model 3 (multiple instruments using *G*_1_ and *G*_2_) had the smallest average bias. The *F*-statistic was greater for the weighted allele score than for the three instrument model (1105 vs. 368) despite having the same average *R*^2^ statistics. This is because the instruments were independent and the weights were derived internally so the weighted score was similar to the linear combination of the instruments derived in the first stage of TSLS.

**Figure 2.**
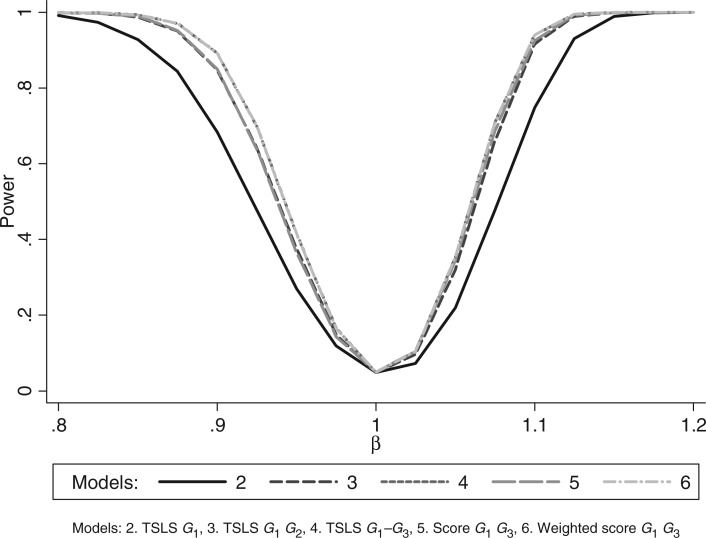
Simulation 1 (non-weak instruments): power curves.

Figure 2 shows that power increased as the number of instruments increased. The power using the unweighted allele score was similar to that using *G*_1_ and *G*_2_ together, while the power using the weighted allele score was the same as using *G*_1_–*G*_3_ together.

### 5.3 Simulation 2: non-weak and weak instruments

Data were simulated with four IVs as follows such that *G*_1_ and *G*_2_ had *F*-statistics greater than 10 and *G*_3_ and *G*_4_ had *F*-statistics less than 10. The variables were simulated as: *G*_1_*_i_* ∼ Bin(2,0.4), *G*_2_*_i_* ∼ Bin(2,0.2), *G*_3_*_i_* ∼ Bin(2,0.2), *G*_4_*_i_* ∼ Bin(2,0.4), and, *U_i_* ∼ *N*(10,1), *X_i_ = *0.1*G*_1_*_i_ + *0.1*G*_2_*_i_ + *0.05*G*_3_*_i_ + *0.05*G*_4_*_i_ + U_i_* and *Y_i_ = βX_i_ + U_i_*. The value of the causal effect of *X* on *Y*, *β*, was set to 1. We monitored the estimates of *β* from the following models:

1. OLS estimate from regression of *Y* on *X*;
2. TSLS estimate using *G*_1_ as the IV;
3. TSLS estimate using *G*_1_ and *G*_2_ as the IVs;
4. TSLS estimate using *G*_1_, *G*_2_, *G*_3_ and *G*_4_ as the IVs;
5. TSLS estimate using an unweighted allele score of *G*_1_ and *G*_2_ as the IV;
6. TSLS estimate using a weighted allele score of *G*_1_ and *G*_2_ as the IV;
7. TSLS estimate using an unweighted allele score of *G*_1_–*G*_4_ as the IV;
8. TSLS estimate using a weighted allele score of *G*_1_–*G*_4_ as the IV.

We used 10 000 replications, each with a sample size of 5 000 observations. We also plotted power curves for testing *β* in the range 0 to 2 (again using 10 000 replications for each value of *β*).

### 5.4 Simulation 2: results

**Figure 3.**
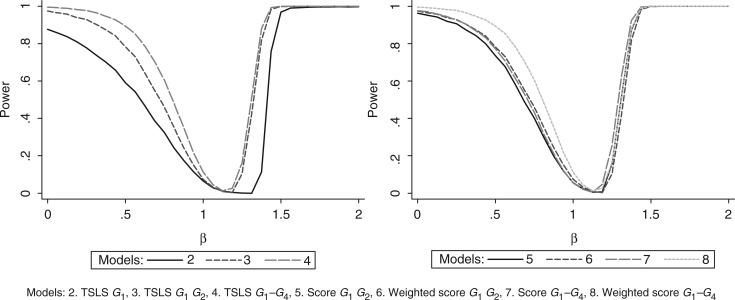
Simulation 2 (non-weak and weak instruments): power curves.

Table 5 shows that models 3 and 6, using the two non-weak IVs as multiple instruments and just these two in a weighted allele score, had the smallest bias. However, models 4 and 8, using all four genotypes as multiple instruments and all four in the weighted allele score, had the smallest MSE and near identical properties to one another, the only difference being that the average *F*-statistic is larger for the weighted allele score due to its smaller model degrees of freedom. Figure 3 shows that models 4 and 8 also had similar power curves and the largest power of the models considered here. These power curves are asymmetric because the distribution of the estimates was negatively skewed in these simulations.

**Table 5.** Simulation 2 (non-weak and weak instruments): results (Monte Carlo standard error in brackets beside each estimate)

Model	Average bias	MSE	Average SE	Coverage	Average *R^2^*	Average F	Av. absolute TSLS/OLS bias ratio
1. OLS	0.990 (0.00001)	0.980 (0.00003)	0.0014 (1.9 E-7)	0 (0)	NA	NA	NA
2. TSLS *G*_1_	−0.047 (0.0025)	0.067 (0.003)	0.237 (0.0015)	0.93 (0.0025)	0.005 (0.00002)	24.92 (0.099)	0.047 (0.003)
3. TSLS *G*_1_ & *G*_2_	0.001 (0.0017)	0.028 (0.0006)	0.164 (0.0006)	0.92 (0.0027)	0.008 (0.00003)	20.99 (0.065)	0.001 (0.002)
4. TSLS *G*_1_–*G*_4_	0.040 (0.0013)	0.020 (0.0003)	0.137 (0.0004)	0.89 (0.0031)	0.011 (0.00003)	13.50 (0.036)	0.041 (0.001)
5. TSLS allele score *G*_1_ & *G*_2_	−0.026 (0.0018)	0.032 (0.0007)	0.172 (0.0006)	0.94 (0.0024)	0.008 (0.00003)	40.99 (0.128)	0.027 (0.002)
6. TSLS weighted allele score *G*_1_ & *G*_2_	0.001 (0.0017)	0.028 (0.0006)	0.164 (0.0006)	0.92 (0.0027)	0.008 (0.00003)	41.99 (0.129)	0.001 (0.002)
7. TSLS allele score *G*_1_–*G*_4_	−0.024 (0.0016)	0.027 (0.0006)	0.160 (0.0005)	0.94 (0.0024)	0.009 (0.00003)	45.91 (0.136)	0.024 (0.002)
8. TSLS weighted allele score *G*_1_–*G*_4_	0.040 (0.0013)	0.020 (0.0003)	0.137 (0.0004)	0.89 (0.0031)	0.011 (0.00003)	54.01 (0.145)	0.041 (0.001)

MSE: mean squared error, SE: standard error, TSLS: two-stage least squares, OLS: ordinary least squares.

## 6 Example revisited: multiple instrument estimates and assessment of missing data

### 6.1 Multiple instrument estimates

The lower half of Table 3 presents IV estimates using two, three and four genotypes and the unweighted and weighted allele scores. The estimated ratios of geometric means were similar, between 1.63 and 1.73, except for the estimate using the unweighted allele score (1.40). Consistent with the simulation studies, the smallest SEs were for the IV estimates using four SNPs and the weighted allele score. For each multiple instrument model, the Sargan over-identification test provides little evidence against the joint validity of the instruments. The Hausman tests suggest that the IV estimates using multiple instruments differ from the OLS estimate.

The SE of the IV estimate using all four SNPs was 0.12, approximately 20% smaller than that of the IV estimate using *FTO* alone (0.16). As expected, given their low first-stage *F*-statistics, inclusion of the *TMEM18* and *GNPDA2* SNPs led only to a small decrease in the SE compared with the multiple instrument model using *FTO* and *MC4R* (0.12 compared with 0.14). The IV estimate using all four SNPs had the largest first stage *R*^2^ and smallest SE.

### 6.2 Assessment of missing data

Table 6 shows IV estimates using the maximum available number of children for each analysis, instead of restricting to children with complete data on all 4 genotypes as in Table 3. Because the sample size increased by only 10–20% for each SNP the SEs of the IV estimates were only slightly smaller than those based on children with complete data. The SE of the IV estimate using all four genotypes as multiple instruments in Table 3 (0.12) was smaller than the SEs of the IV estimates using all available data using one, two and three instruments in Table 6.

**Table 6.** IV estimates of the effect of fat mass on bone mineral density (BMD) using all available data^a^

SNPs used as instrumental variable	*N*	First stage regression coefficient (95% CI)	First stage *R^2^*	First stage * F*-statistic	Ratio of geometric mean BMD^b^ (95% CI)	SE of estimate (log scale)	Hausman test *p*-value	Sargan test * p*-value
OLS	5509	NA	NA	NA	1.22 (1.18, 1.25), *p* < 0.001	0.014	NA	NA
IV: SNP(s) used as IV								
*FTO*	5091	0.12 (0.08, 0.15)	0.0088	45.35	1.41 (1.05, 1.89), *p* = 0.023	0.15	0.320	NA
*MC4R*	5412	0.09 (0.05, 0.13)	0.0037	19.95	2.42 (1.42, 4.12), *p* = 0.001	0.27	0.002	NA
*TMEM18*	5323	−0.06 (−0.11, −0.02)	0.0013	6.99	2.17 (0.92, 5.12), *p* = 0.077	0.44	0.130	NA
*GNPDA2*	5303	0.05 (0.01, 0.08)	0.0013	6.90	0.92 (0.42, 2.01), *p* = 0.84	0.40	0.463	NA
*FTO*, *MC4R*	5007	NA	0.0125	31.61	1.60 (1.24, 2.07), *p* < 0.001	0.13	0.029	0.221
*FTO*, *MC4R*, *TMEM18*	4881	NA	0.0138	22.75	1.69 (1.32, 2.17), *p* < 0.001	0.13	0.006	0.227

a Analyses adjusted for height and height squared.

b For a 1 unit increase in *z*-score of age and gender standardised fat mass.

## 7 Discussion and conclusion

Mendelian randomisation studies using genetic variants as instruments can control for unmeasured confounding and reverse causation, which can bias results from standard epidemiological analyses. However, population stratification, LD and pleiotropy can all affect the validity of the IV assumptions underlying Mendelian randomisation analyses. Obtaining similar IV estimates from separate independent instruments provides evidence against the presence of bias from pleiotropy and LD, though not bias from population stratification. In our example there was no evidence that the estimates for each instrument differed from each other (based on the over-identification test), providing some reassurance that bias from pleiotropy and LD is unlikely. However, we acknowledge in this example our power to detect differences between the estimates was limited.

Mendelian randomisation analyses require large sample sizes unless the instrument is strongly related to the risk factor (phenotype) of interest. Use of multiple genetic variants as IVs increases the power of such analyses and facilitate tests of the IV assumptions that are not possible in single instrument analyses (such as the test of over-identification). However, inclusion of instruments that explain only a small proportion of the variability in the phenotype can increase finite sample bias of IV estimates. We have limited our consideration to the linear IV model. Non-linear models that naturally arise for discrete outcomes require different treatment.^11^

Our illustrative Mendelian randomisation analysis confirmed a positive causal effect of adiposity (fat mass) on BMD, in line with previous research ^31^ and suggested that the size of this effect was larger than that estimated by ignoring unmeasured confounding and using ordinary least squares, based on the Hausman endogeneity test. The SE of the IV estimate decreased by around 20% using all four genotypes, compared with the SE of the IV estimate using only the genotype with the strongest effect on risk factor. Such a reduction in SE corresponds to a 56% increase in sample size.

With increasing availability of multiple genetic variants associated with the same risk factor or disease outcome, it is becoming common for genetic association studies to report associations with allele scores.^64,65^ Before an allele score is used as an IV the joint validity of the SNPs should be assessed using an over-identification test. The weights used in weighted allele scores may be internal or external to the study: when internally estimated the single degree of freedom used in the *F*-statistic for instrument strength may not be appropriate. In their simulations Pierce et al. ^13^ used external weights based on the true effect of the genotypes on the phenotype: such weights should be taken from the overall available evidence. They concluded that unweighted and weighted allele scores, using these external weights, decreased bias when compared to the traditional multiple instruments approach, but that they had less power than the multiple instruments approach. In our simulations, models including all instruments, either as multiple instruments or in a weighted allele score, had the greatest power and lowest MSE but not the smallest bias. Based on these results the use of allele scores as IVs can represent a good trade off in terms of lower bias but possibly less precision compared to the TSLS estimator. It has been shown that for larger numbers of IVs, with differing effect sizes, it is better to use a weighted allele score.^13^

Another consequence of the large number of genetic variants that are being indentified in GWAS in relation to particular phenotypes is that it is possible to generate many independent combinations of such variants and from these many independent IV estimates of the causal effect of a risk factor on a disease outcome. These independent estimates will not be plausibly influenced by any common pleiotropy or LD-induced confounding, and therefore if they display consistency would provide strong evidence against the notion that reintroduced confounding is generating the effect.^67,68^

There are typically missing data on each genetic variant, due to failure of genotyping or ambiguous genotype allocation. Thus in multiple instrument analyses, missing genotype data can offset improvements in power compared with single instrument analyses. It may be reasonable to assume that the mechanism causing genetic data to be missing is independent of a particular analysis of interest, so this may not be a cause of bias. There is scope for methodological research into multiple imputation strategies for IV estimators. It might also be possible to impute missing data for single SNPs by exploiting the LD structure between SNPs in LD with them, as is common in GWAS.^69^ In the ALSPAC study, maternal genotypes are available, which could also be used to impute missing offspring genotypes.

In conclusion, the use of multiple genetic instruments increases the statistical power of Mendelian randomisation analyses and provides opportunities to test IV assumptions.
